# Effects of the addition of short straight steel fibers on the strength and strains of high-strength concrete during compression

**DOI:** 10.1038/s41598-024-57574-1

**Published:** 2024-03-24

**Authors:** Maciej Kaźmierowski, Roman Jaskulski, Michał Drzazga, Marek Nalepka, Michał Kordasz

**Affiliations:** 1 Department of Civil Engineering, Faculty of Environmental Engineering and Geodesy, Wrocław University of Environmental and Life Sciences, 50-365 Wrocław, Poland; 2 Department of Mechanics and Structural Engineering, Faculty of Civil Engineering and Architecture, Opole University of Technology, 45-061 Opole, Poland; 3 Department of Graphics, Computer Vision and Digital Systems, Faculty of Automatic Control, Electronics and Computer Science, Silesian University of Technology, 44-100 Gliwice, Poland

**Keywords:** Civil engineering, Composites

## Abstract

The article presents the effect of the addition of short straight steel fibers on the behavior of high-strength concrete (HSC) under compression (σ–ε curves). Deformations of cylindrical samples were measured simultaneously with the use of linear variable differential transformers (LVDT), strain gauges and the method of digital image correlation (DIC). The study showed that as the content of short straight steel fibers increases, both the composite compressive strength (f_c_) and strains (ε_0_), which correspond to the stress equal to the compressive strength, increase as well. To a lesser extent, the effect of short straight fibers on the descending part of the σ–ε curve was observed. An increase in the density and toughness ratio of the compressive strength of high-strength concrete with fibers compared to concrete without fibers was also observed. Moreover, compressive strength of the composite was estimated using the ultrasonic method. Based on the obtained results, a statistical analysis and an estimation of parameters f_c_ and ε_0_ were carried out, and an analytical model was proposed to describe σ–ε relationship for HSC reinforced with short straight fibers under compressive loading. The results obtained for compressed fiber-reinforced concrete were compared with data available in literature.

## Introduction

Strength properties of concrete are most often determined by specifying its compressive strength^1^. High-strength concrete (HSC) in comparison to plain concrete, is characterized by higher strength and lower permeability, which translated into lower porosity. HSC also exhibits different behavior under compressive loading than plain concrete due to differences in their composition and microstructure^1^.

One of the ways to increase strength and strain susceptibility of HSC concrete under compression (to reduce brittleness) is to add steel fibers^2,3^. Volumetric fiber content up to 1.5% in HSC (V_f_) generally increases compressive strength by about 10–20% compared to HSC without fibers^4,5^. There are also studies which show no effect of fibers on compressive strength, or even its reduction^6–9^. With increasing fiber content (V_f_) in the composite, there is also an increase in the strain at maximum compressive stress^5,7–11^, while the effect of fiber addition on the rising part of the stress–strain curve (σ–ε) is small and clearly noticeable for the descending part of the σ–ε graph^4^. The descending part of the σ-ε curve is an important element in nonlinear analysis, the design of fiber reinforced compressed elements^12^, describes the evolution of internal damage in concrete at the macroscopic level under compressive loading (constitutive model of concrete) and provides a starting point in determining the load-bearing capacity and deformations of concrete^2^.

It is worth noting that increasing the ultimate strain of HSC under compression as well as the section curvature (e.g. by using steel fibers) increases the possibility of redistribution of internal forces in the structure, which is beneficial to its safety^13^. Adding fibers to concrete significantly increases its tensile strength, fracture energy, impact resistance, fire-resistance and durability^14–18^. Therefore, this type of composite is being increasingly used in construction (including beams, slabs, columns and walls)^19^. Steel fibers with hooked ends demonstrate greater efficiency compared to other forms of fibers due to their higher resistance during pull-out from the matrix, so they are widely used in construction^19^. However, other types of fibers—including straight ones—are also used to improve the performance of concrete^14,20^.

Although the effect of steel dispersed reinforcement on the strength and deformation of concrete under compression has been the subject of numerous studies^5,8,10,11,21–23^, the effect of short straight steel fibers on the compressive strength of HSC—and particularly on its deformation—has not been widely discussed. This research focuses on filling this gap, hence the article presents experimental and theoretical studies of HSC reinforced with short steel fibers under compressive loading. The data and findings of this article may provide researchers with valuable information for further study of such composites.

## Materials and methods

### Experimental program

The program of the study included preparing three batches of samples (6 samples in each batch, designated as A, B and C) of HSC with different steel fiber content (V_f_). Tests were then carried out, including the determination of σ-ε curves during compression, compressive strength (also by ultrasonic method), strains corresponding to stress equal to compressive strength, toughness ratio and density of the composite.

### Materials

**Figure 1:**
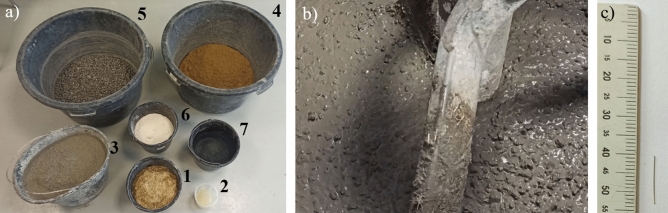
(**a**) View of the prepared ingredients of the concrete mix for one C-series batch (numbering: 1-steel fibers; 2-superplasticizer; 3-cement; 4-sand; 5-crushed granite; 6-lime powder; 7-water); (**b**) view of the mixture in the mixer; (**c**) length of fibers used.

The tests were conducted on cylindrical samples of 150 × 300 mm^24^. The concrete mix was designed assuming strength grade C90/105^25^. For the mix, the following were used: Portland cement CEM-I 52,5N (ρ = 3050 kg/m^3^); natural washed aggregate of 0–2 mm fraction (ρ = 2550 kg/m^3^); crushed granite aggregate of 2–8 mm fraction (ρ = 2660 kg/m^3^); superplasticizer admixture based on polycarboxylate (ρ = 1100 kg/m^3^) and lime powder with CaCO_3_ > 94% and grain fraction < 0.075 mm at least 80%, (ρ = 2650 kg/m^3^). The water-cement ratio of the mix was 0.29. The composition of the concrete mix of each batch is shown in Table 1. Figure 1 shows the ingredients of the concrete mix for one C-series batch.

**Table 1:** Composition of the concrete mix for each batch.

Ingredient number*	Ingredient	Unit	Batch		
			A	B	C
1	Steel fibers	kg/m^3^ %	0	58.87 0.75	117.75 1.5
2	Superplasticizer	kg/m^3^	3.2	4.4	5.1
3	Cement—CEM I 52.5R	kg/m^3^	637.5		
4	Sand 0–2 mm	kg/m^3^	625		
5	Crushed granite 2–8 mm	kg/m^3^	725		
6	Lime powder	kg/m^3^	150		
7	Water	kg/m^3^	184.9		

*The ingredient number corresponds to the numbering in Fig. 1a.

Straight smooth cold-drawn wire fibers (high-carbon steel) were used, 13 mm in length, 0.2 mm in diameter (fiber slenderness λ = 13/0.2 = 65), with density of 7850 kg/m^3^, elastic modules of 200 GPa and tensile strength of 2000 MPa. All ingredients of the mix were dosed by weight with an accuracy of up to 1%. The order of dosing the ingredients is described below. First, fine aggregate was mixed with coarse aggregate, then cement and lime powder were added. After the dry ingredients had been mixed, water was added, and later superplasticizer. The last ingredient added to the mix were steel fibers. Total mixing time was approximately 8 min. In order to obtain the same consistency of the concrete mix for all batches, while keeping the w/c ratio constant (mitigating workability deterioration in the mix caused by the presence of fibrous inclusions), test batches were made, and on their basis the optimal content of superplasticizer in each batch of the composite (A, B and C) was determined. Consistency testing was carried out using the drop-cone method^26^. The liquidity index of the mixes ranged from 190 to 200 mm (consistency class S4). The test samples were stored and cured for in accordance with the standards^27^.

### Research methodology

**Figure 2:**
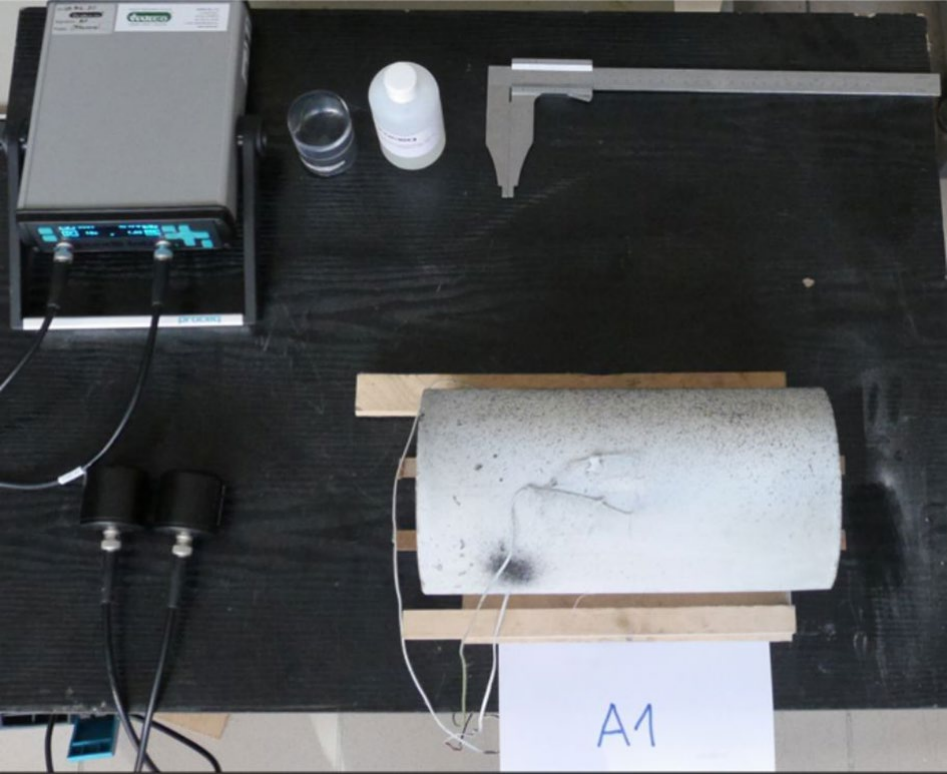
Experimental setup for testing samples by ultrasonic method.

**Figure 3:**
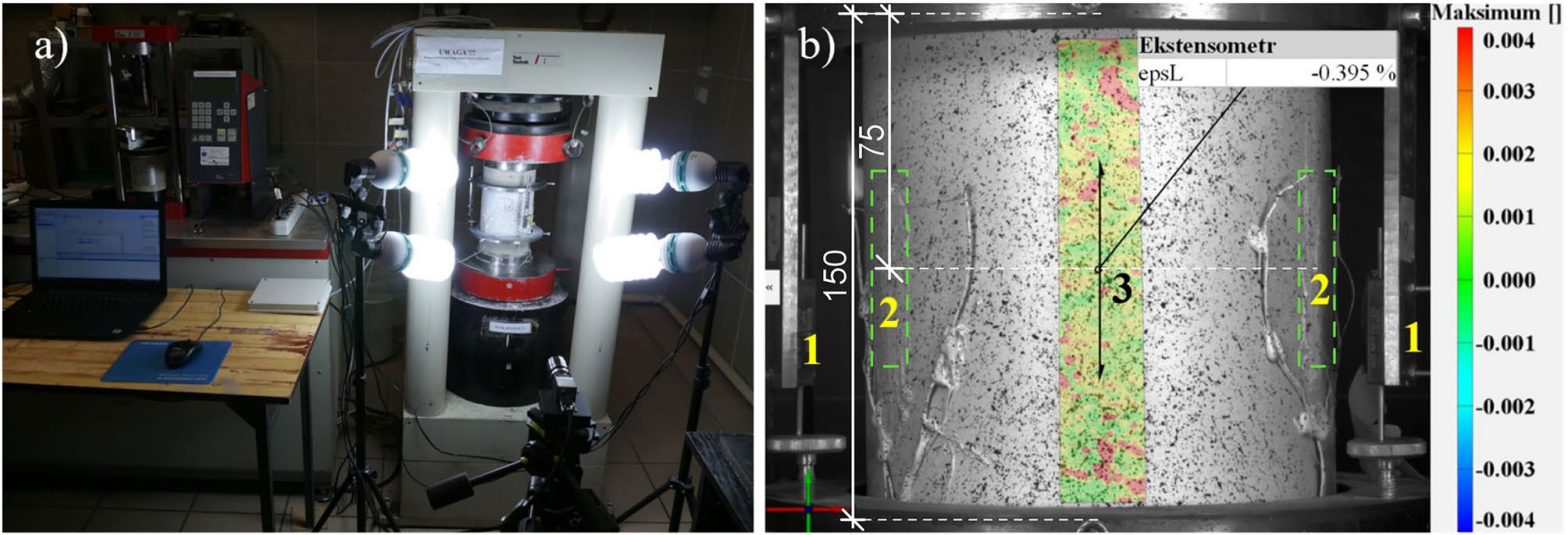
Experimental setup for samples in compression: (**a**) general view; (**b**) view of the location of the measuring elements together with the map of vertical strain according to DIC for sample B4 before destruction (1-LVDT sensors, 2-strain gauges, 3-virtual extensometer).

First, all samples were subjected to density testing with the hydrostatic method. Next, ultrasonic tests were carried out in order to compare the results of the acoustic wave propagation speed in the samples with the results of their compressive strength (Fig. 2). During tests, touch probes were placed at the bases of the cylinders. The following assumptions were made: frequency of the transducer of 54 kHz, pulse width of 9.3 μs, gain of the receiver of 1000x, trigger voltage of 500 V and intermittent wave transmission. Compressive strength tests of the samples were carried out in accord with the standard^28^ in a hydraulic press with a maximum pressure of 3000 kN (Fig. 3a). The surfaces of the samples were leveled using the sand overlay method before testing. The samples were then centered and preloaded. Loading was implemented by controlling the displacement of the piston at a value of 0.07 mm/min, until the destroyed of the sample. This value was adopted based on the analysis of papers^5,21,28,29^ and technical capabilities of the testing equipment. The strains in samples were recorded using three LVDT sensors, located at the side of the cylinder every 120° and attached to steel rings, which were fastened with three contoured bolts to the concrete. The arrangement of sensors and rings was fixed symmetrically with respect to the horizontal axis of symmetry of the specimen. The measurement base of the measured strains of the specimen (for LVDT sensors) was 150. The sensors had a resolution of 1 μm, and a measurement range of 15 mm. In addition, strains were measured using three foil strain gauges with a measuring base of 60 mm and a resistance of 120 Ω, placed at locations determined by the projections of LVTD on the side surface of the cylindrical sample and at the midpoint of its height. Using the digital image correlation method (DIC)^30,31^, linear strains were also recorded with a virtual extensometer with a base of 70 mm which was defined at mid-height of the side, symmetrically to strain gauges 1 and 2 (Fig. 3b). A camera with a resolution of 4096 × 3000 pixels, i.e. 12 Mpix, and a shooting rate of 12 Hz was used for the study. The basic version of the system allows to measure displacements and strains in plane. Therefore, strain analysis was limited to the use of the mentioned extensometer. The results were computer recorded with time synchronization. All the aforementioned tests were carried out 28 days after concreting.

## Ethical approval

This article does not contain any studies with human participants or animals performed by any of the authors.

## Results

### Material constants of the composite

Table 2 shows the specifications of the samples used and the results of the tests: compressive strength (f_c_); strain measured with LVDT sensors (ε_0_^LVDT^), strain gauges (ε_0_^GAUGE^) and virtual extensometer (ε_0_^DIC^); wave velocity (v), toughness ratio (TR) and density (ρ). The strains ε_0_^LVDT^, ε_0_^GAUGE^ i ε_0_^DIC^ correspond to the maximum compressive stress. Toughness ratio TR is determined by Eq. (1) (the area under the σ-ε curve was calculated by numerical integration using the rectangle method). For each batch, arithmetic mean (x), standard deviation (s) and coefficient of variation (v) of measured values were also determined. The strains ε_0_^LVDT^ and ε_0_^GAUGE^ represent the value averaged from the readings of the three gauges.

1 $$TR=\frac{ED}{0.015{f}_{c}}$$

where:

**Table 2:** Sample tests results (the description of the designations used in the text).

	f_c_	ε_0_^LVDT^	ε_0_^GAUGE^	ε_0_^DIC^	v	TR	ρ
s. No	MPa	‰			m/s	-	kg/m^3^
A1	97.2	3.34	3.25	3.74	4660	0.116	2304
A2	101.6	3.12	3.19	3.30	4702	0.105	2313
A3	97.7	3.29	3.11	2.98	4688	0.097	2304
A4	94.8	3.00	3.06	2.99	4710	0.100	2306
A5	87.5	3.14	2.85	3.38	4690	0.094	2264
A6	93.0	3.02	3.02	3.09	4679	0.096	2288
**x**	**95.3**	**3.15**	**3.08**	**3.24**	**4688**	**0.102**	**2297**
**s**	**4.8**	**0.14**	**0.14**	**0.29**	**17.60**	**0.01**	**17.91**
**ν**	**5.0%**	**4.5%**	**4.6%**	**9.0%**	**0.4%**	**7.9%**	**0.8%**
B1	107.7	3.40	3.29	3.11	4800	0.117	2363
B2	79.9	2.96	2.50	2.73	4772	0.091	2290
B3	112.8	3.86	3.61	3.61	4856	0.118	2368
B4	102.9	3.35	3.20	3.95	4754	0.104	2371
B5	109.3	3.64	3.54	4.18	4753	0.123	2361
B6	114.1	3.68	3.81	4.13	4753	0.128	2364
**x**	**104.4**	**3.48**	**3.32**	**3.62**	**4781**	**0.114**	**2353**
**s**	**12.7**	**0.32**	**0.46**	**0.59**	**40.90**	**0.01**	**30.99**
**ν**	**12.1%**	**9.1%**	**13.8%**	**16.2%**	**0.9%**	**11.9%**	**1.3%**
C1	117.1	3.82	3.58	3.13	4893	0.122	2410
C2	117.5	3.95	3.48	3.91	4897	0.114	2405
C3	110.2	3.64	3.56	3.32	4826	0.128	2414
C4	115.5	3.60	3.57	4.34	4783	0.127	2403
C5	117.9	3.88	3.58	3.78	4904	0.132	2415
C6	113.8	3.59	3.47	4.38	4895	0.111	2409
**x**	**115.3**	**3.75**	**3.54**	**3.81**	**4866**	**0.122**	**2409**
**s**	**2.94**	**0.16**	**0.05**	**0.51**	**49.93**	**0.01**	**4.76**
**ν**	**2.6%**	**4.2%**	**1.4%**	**13.5%**	**1.0%**	**6.7%**	**0.2%**

ED—the energy absorption capacity by fiber-reinforced concrete or surface area under the σ–ε curve [MPa]. The value of 0.015 was proposed by Ezeldin and Balaguru^11^.

### Stress–strain relationship

**Figure 4:**
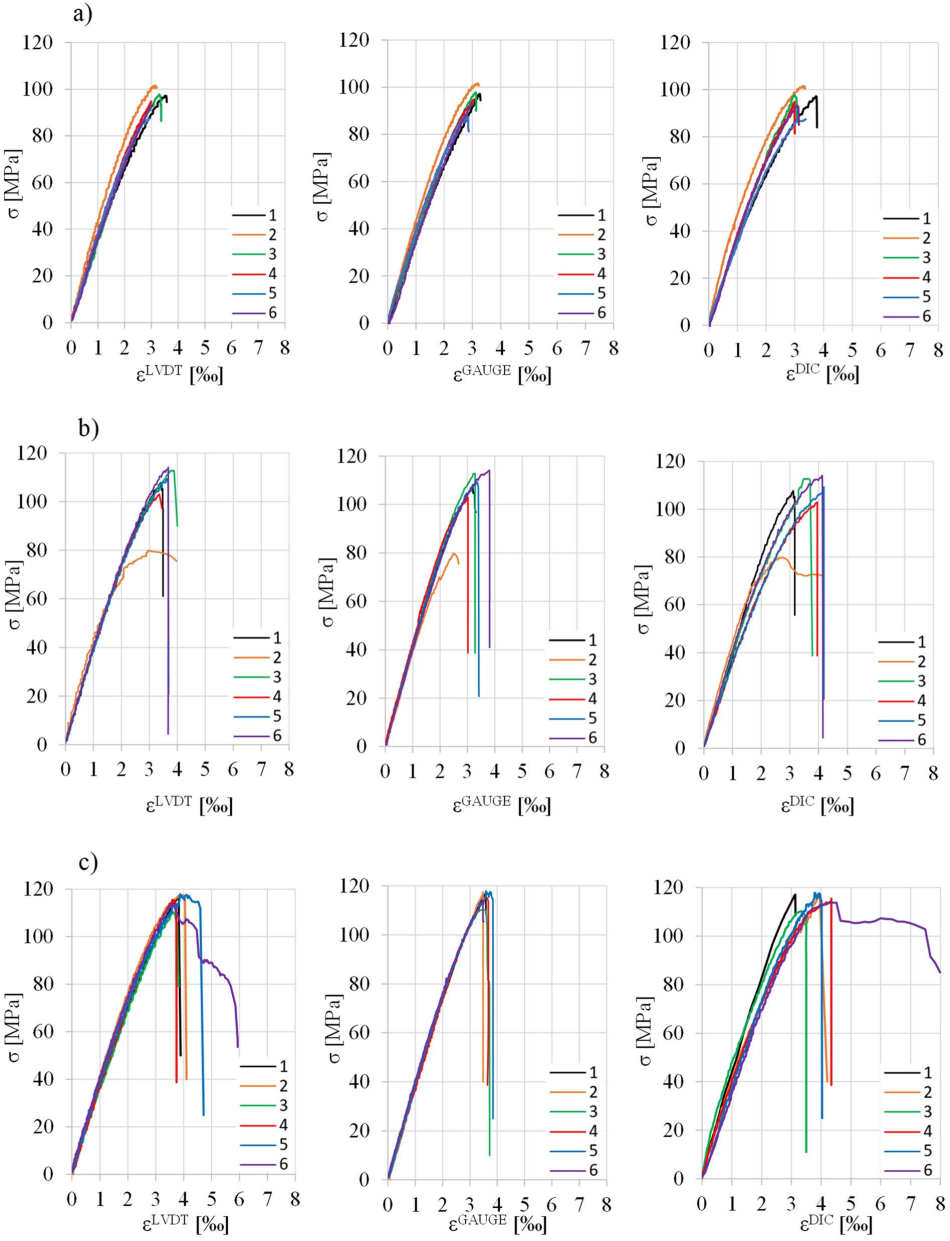
σ-ε curves of the samples in compression, determined by three methods (description in text): (**a**) A-series; (**b**) B-series; (**c**) C-series.

**Figure 5:**
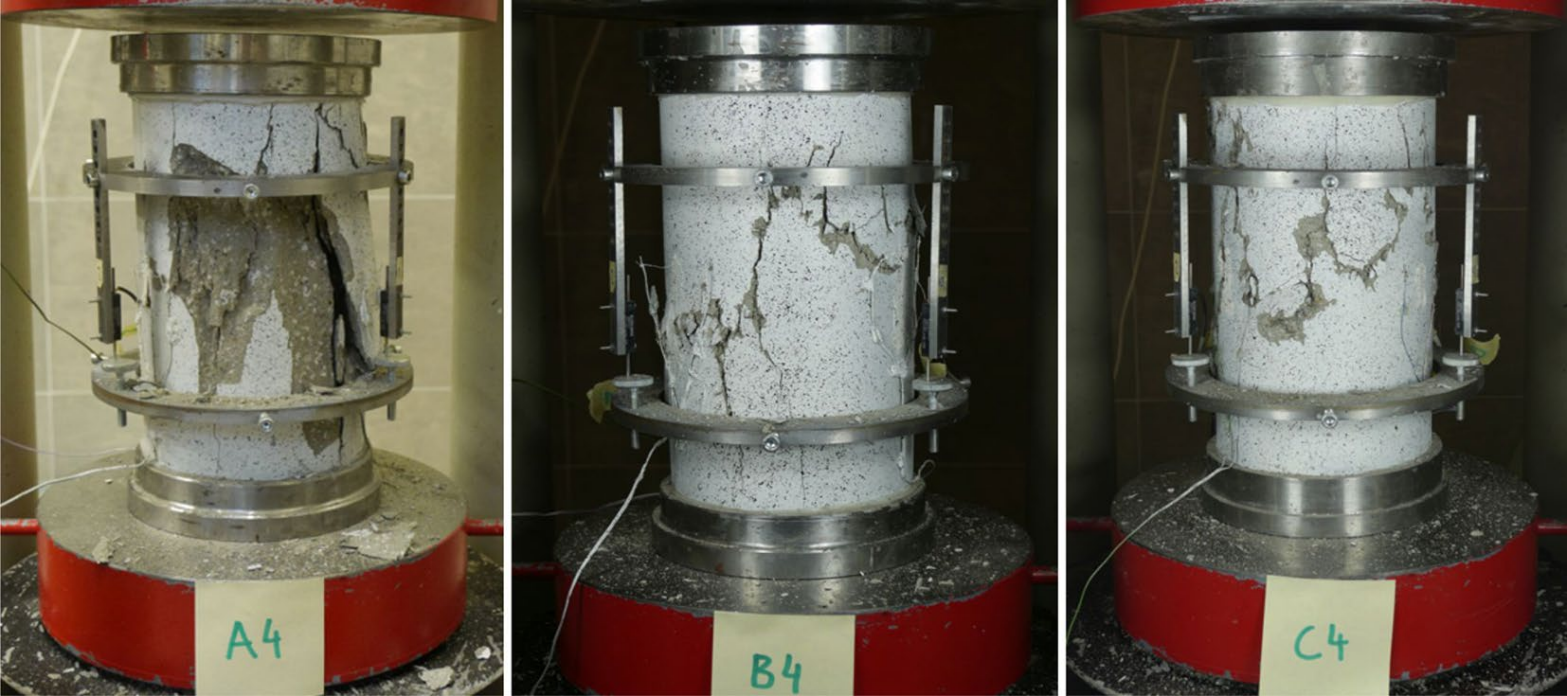
Images of damaged samples (from left: sample A4, B4 and C4).

Figure 4 shows σ–ε curves of samples with different steel fiber content (batches A, B and C). The curves were determined on the basis of mean values from LVDT readings (ε^LVDT^), as well as the mean from the strain gauges values (ε^GAUGE^). The strains ε^DIC^ were measured at one place (see Fig. 3b). Figure 5 show example images of the damaged samples.

## Discussion

### Material constants of the composite

The volume proportion of steel fibers (V_f_), which was 0.75% or 1.5% in batches B and C, resulted in an increase of 9.6% and 21% in compressive strength (f_c_) compared to batch A (control), respectively. The coefficient of variation of f_c_ strength of the individual batches was 5.0%; 12.7% and 2.9%, respectively (Table 2). The results obtained are confirmed by studies that report an increase in the compressive strength of high-strength fiber-reinforced concrete from 10 to 20% with the addition of fibers 0–2%^6,32,33^.

**Figure 6:**
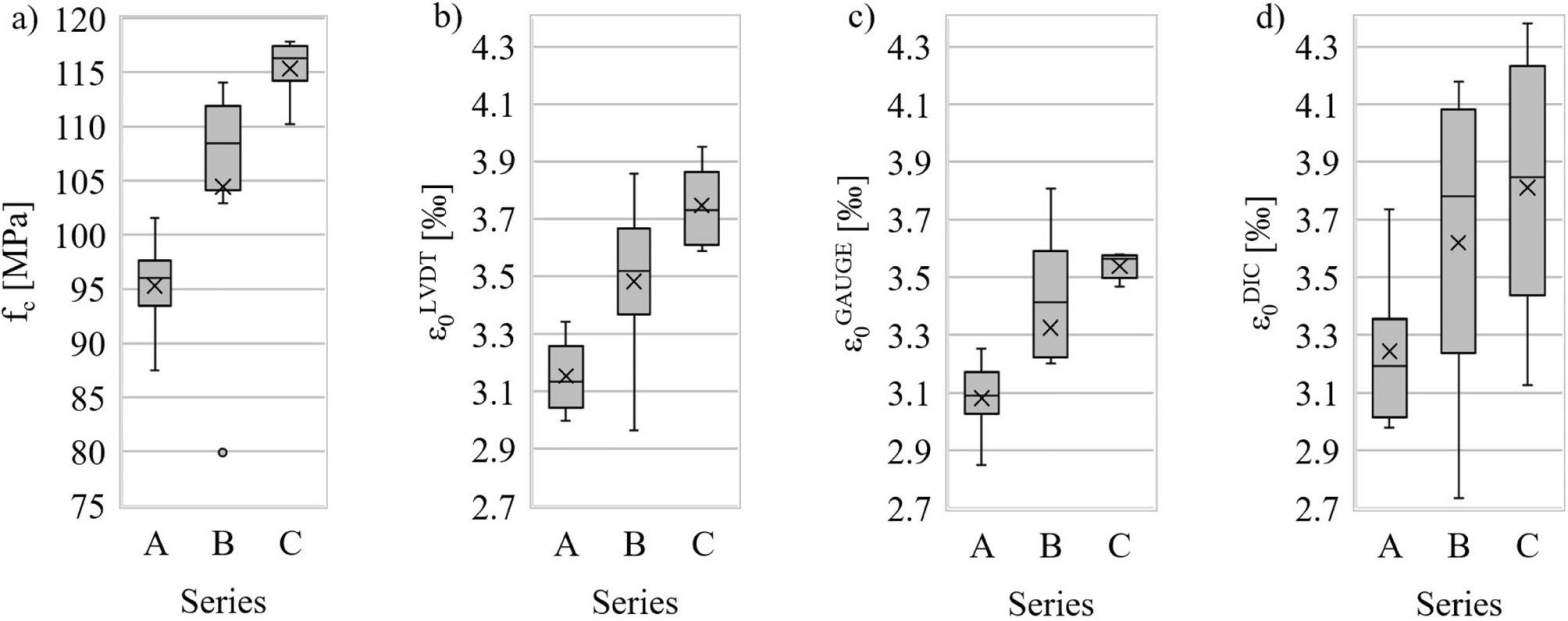
Box plot of: compressive strength ((**a**); strain ε_c_ measured with LVDT sensors ((**b**), strain gauges ((**c**) and DIC method ((**d**).

Selected statistics of strength distribution f_c_ and strains ε_0_ are shown in a box plot (Fig. 6). These are the positions of the arithmetic mean, median, first and third quartiles, outlying values, maximum and minimum values (including the median). The outlying observation visible on the graph (79.9 MPa—sample B2), was further confirmed with the Grubbs statistical test (in the remainder of the paper, regression analyses and σ–ε comparisons were performed excluding the outlier). Based on the standard^34^, samples from batch A were assigned a concrete grade of C90/105, whereas those from batches B and C—were assigned a grade of C100/115.

**Figure 7:**
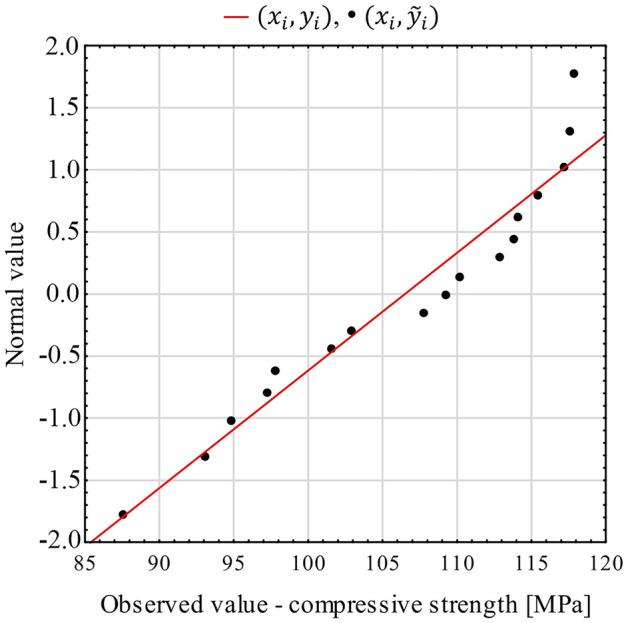
Normality diagram of compressive strength (explanation: ${y}_{i}={(x}_{i}-\overline{x})/s$; ${\widetilde{y}}_{i}={\phi }^{-1}({u}_{i})$; ${p}_{i}=(3i-1)/(3n+1)=\phi ({u}_{i})$; ${x}_{i}$—compressive strength results, ordered in ascending order; $\overline{x}$— arithmetic mean; s—standard deviation; $\phi ({u}_{i})$—standard normal distribution cumulative distribution function for compressive strength; ${p}_{i}$—cumulative frequencies; *i*—index in ascending series; *n*—number of observations).

In order to determine the significance of differences between the mean values of the compressive strength tests of each batch, one-way analysis of variance (ANOVA) was performer using the Statistica software, taking the fiber-reinforcement ratio (V_f_λ) as a qualitative predictor. The assumptions of the ANOVA tests in the form of normality of distribution within groups and homogeneity of variances were met. The normality of distribution is shown in Fig. 7 and confirmed by the Shapiro–Wilk test (test value *p* = 0.13 and is greater than the accepted level of significance α = 0.05). Homogeneity of variance was checked with Levene’s test (*p* = 0.62 > α). The ANOVA analysis of variance test shows that the p value is lower than the accepted level of significance, so there is a basis for rejecting the null hypothesis. This means that mean compressive strength in batches A, B and C is significantly different (Table 3). Analogous analyses and conclusions apply to the differences between the mean values of different batches as regards: strain ε_0_, ultrasonic wave velocity v and toughness ratio TR (Table 3). In all cases, *p* <  < α was obtained with the exception of differences in mean strain ε_0_^DIC^ (*p* slightly greater than α).

**Table 3:** Results of ANOVA calculations of material constants of the composite.

Characteristic	SS_E_ Effect	MS effect	SS effect	MS effect	F	*p*
f_c_	1260.7	630.35	236.1	16.9	37.4	2.4 × 0^−6^
ε_c_^LVDT^	1.12	0.56	0.40	0.028	19.8	8.4×10^−5^
ε_c_^GAUGE^	0.74	0.37	0.36	0.025	14.6	3.8 × 10^−4^
ε_c_^DIC^	1.22	0.61	2.53	0.180	3.4	6.2 × 10^−2^
v	85689.6	42844.8	24393.6	1742.4	24.6	2.6 × 10^−5^
TR	1.4$\cdot$10^−3^	7.1$\cdot$10^−3^	9.6$\cdot$10^−4^	6.9$\cdot$10^−5^	10.4	1.7 × 10^−3^

SS_E,_ sum of squares of deviation of individual group means from the global mean; MS_E_ = SS_E_/d_f1_; SS_B_, sum of squares of deviation of sample results from their group means; MS_B_, SS_B_/d_f2_; F, test value; *p*, test probability level; d_f1_, d_f2,_ number of degrees of freedom for inter- and intra-group variability.

The addition of fibers increased the strain ε_0_ corresponding to the maximum compressive stress (this was confirmed by all strain measurement methods). For batch B, the increase was 7–12%, while for batch C it was 14–19%.

**Figure 8:**
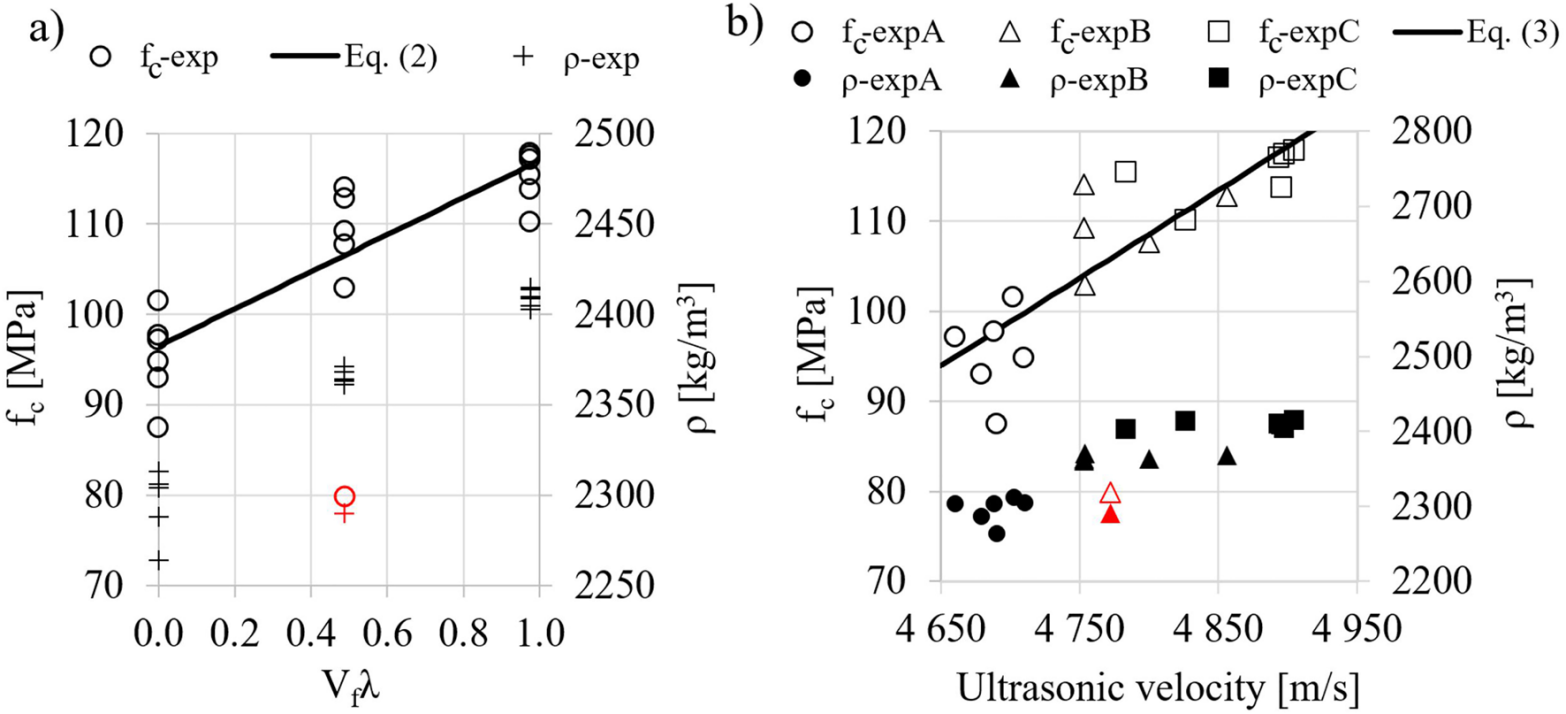
Compressive strength (f_(**c**)_ and density (ρ) as a function of: degree of fiber reinforcement ((**a**) and ultrasonic wave velocity ((**b**); (red color indicates outliers that were not included in the regression analysis).

Figure 8a shows the results of compressive strength and density of the composite depending on the degree of fiber reinforcement (V_f_λ) and the regression equation Eq. (2) with the determination coefficient R^2^ = 0.79. The standard error of the slope coefficient was 2.62. Based on t-Student statistic, for the assumed level of significance, a significantly statistical structural parameter of the model was obtained (*p* <  < α).

2 $${f}_{c}=20.54{V}_{f}\lambda +{f}_{c}{\prime}$$

where ${f}_{c}{\prime}$—compressive strength of HSC without fibers.

Figure 8b shows the relationship between compressive strength and ultrasonic wave velocity results (also the relationship for composite density). A linear equation was proposed to estimate the compressive strength as a function of wave speed Eq. (3) with R^2^ = 0.75. The standard error of estimators of the equation Eq. (3) was 0.0147 for the slope coefficient and 70.3 for the intercept (statistically significant model parameters).

3 $${f}_{c}=0.0974v-358.8$$

The regression line shows a general trend that an increase in the wave speed of the sample corresponds to its higher compressive strength. The use of the ultrasonic method can be useful for evaluating the strength of HSC concretes reinforced with short steel fibers. Despite the good fit of the data to models 2 and 3, it is important to keep in mind their limitations in application, since they were determined from experimental data obtained according to the adopted test method. The use of polynomial instead of linear approximation in models 2 and 3 increases the value of the coefficient of determination by about 5%, but the model parameters are statistically insignificant.

**Figure 9:**
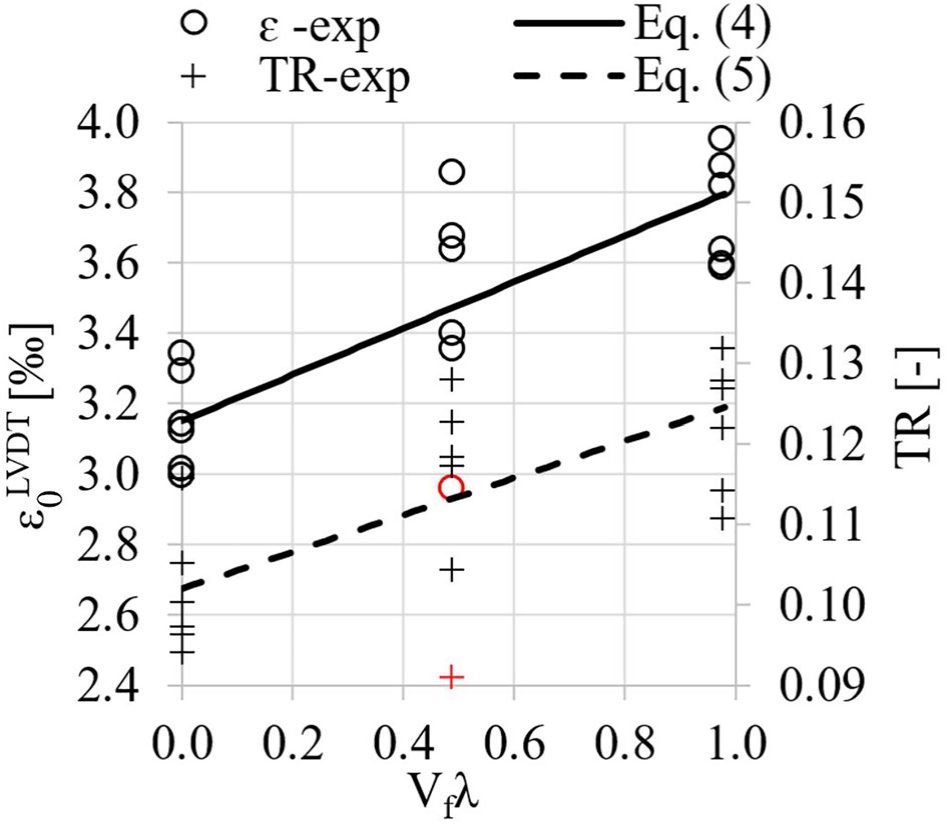
Strains ε^LVDT^ corresponding to maximum compressive stress and toughness ratio (TR) as a function of the degree of fiber reinforcement (red color indicates outliers that were not included in the regression analysis).

Figure 9 shows the dependence of strain ε_0_^LVDT^ and toughness ratio TR on the degree of fiber reinforcement V_f_λ and the corresponding regression equations Eq. (4 i 5), for which the coefficient R^2^ was 0.69 and 0.54. The parameters of the models are statistically significant (standard errors of the values of the slope coefficients were 0.066 and 0.0032).

4 $${\varepsilon }_{0}={\varepsilon }_{0}{\prime}+0.66{V}_{f}\lambda$$

5 $$TR=TR{\prime}+\mathrm{0,023}{V}_{f}\lambda$$

where ${\upvarepsilon }_{0}^{\mathrm{^{\prime}}}$—strains of concrete without fibers in [‰], $TR{\prime}$—toughness ratio of concrete without fibers.

### Stress–strain relationship

Analyzing the results from Fig. 4, the course of σ–ε curves may be divided into three phases. Phase I—approximately linear-elastic σ–ε relationship (the slope of the curves of all samples remains practically the same when the stresses are not large); phase II—nonlinear σ–ε relationship before reaching the maximum stress σ_max_ (the development of internal micro-damage of the composite and its non-elastic properties are the reason for the nonlinear shape of the graph at higher stress) and phase III—after exceeding the stress σ_max_, the curves drop rapidly, with the rate of descent for samples with fibers being different and slightly smaller than those of concrete without fibers. The fiberless concrete samples underwent explosive destruction after the σ_max_ stress was reached (Fig. 5), while fiber-reinforced ones showed the ability to carry a small residual stress (Fig. 4), which was preceded by a sharp drop in stress (with a marked difference in the case of samples C5 and C6). In other words, the energy-absorbing capacity of fiber-reinforced samples compared to samples without fibers is relatively low and less than the difference observed in studies of similar subject matter^5,8,11,21,22,29^.

**Figure 10:**
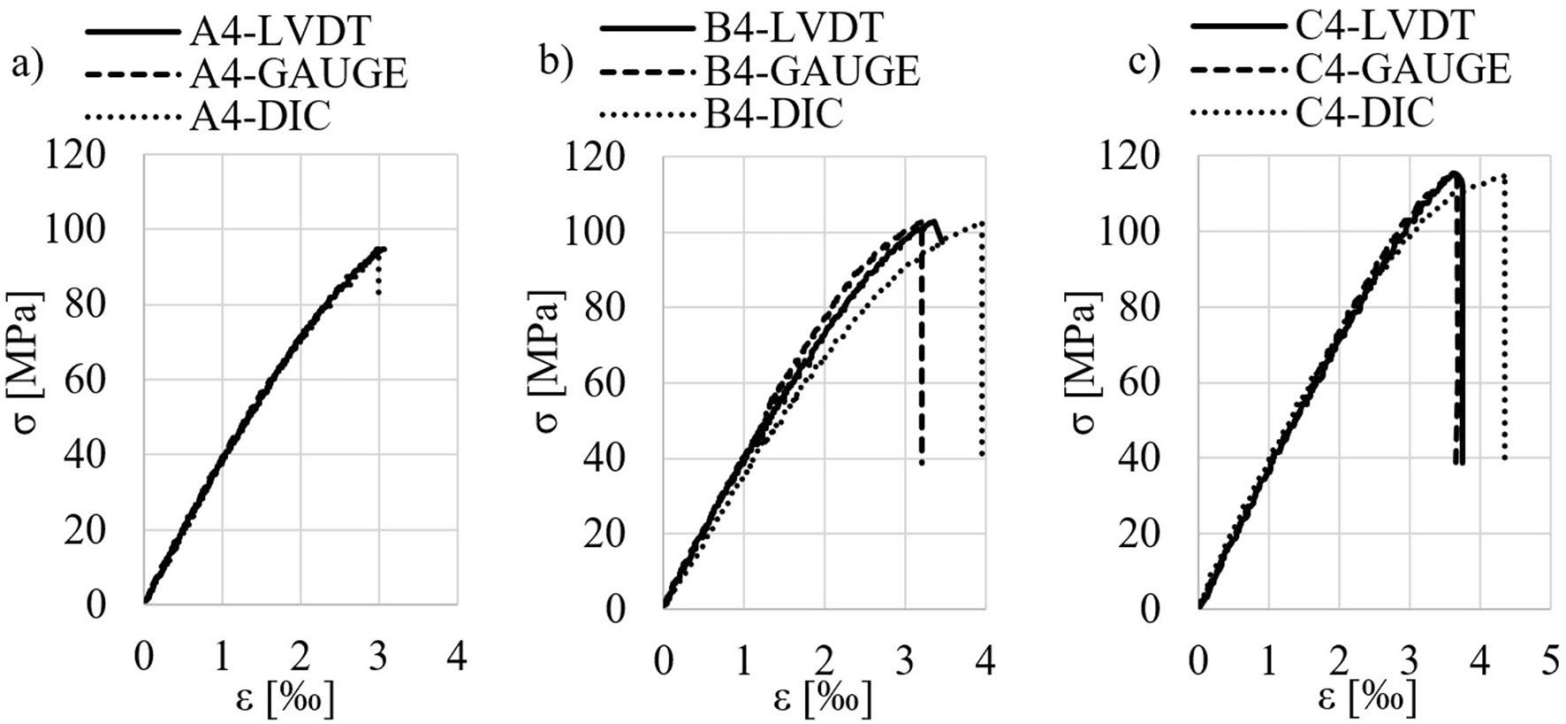
σ-ε relationships in compression obtained from LVDT sensors, strain gauges and the DIC method for samples: (**a**) A4; (**b**) B4 and (**c**) C4.

It should be noted that in literature mentioned above, other than short straight fibers for concrete were used (hooked, corrugated). Despite the satisfactory agreement of the results, i.e. σ-ε curves over the entire load range for the three strain measurement methods (for example, for selected samples they are shown in Fig. 10), it is worth noting that the characteristics of σ-ε curves of compressed samples strongly depend on the structure of dispersed fiber reinforcement, which is described by mechanical efficiency. It determines the interaction of individual fibers in the concrete, enabling it to inhibit cracking. Mechanical efficiency depends on: the slenderness (λ) and shape of the fibers, the volumetric proportion of the fibers in the composite (V_f_), the spatial distribution of the fibers in the concrete, and the adhesion of the fibers to the cement matrix, resulting from adhesion, friction and mechanical anchorage^35^. The adhesion of smooth, straight fibers to the matrix is due only to adhesion and friction, while fibers deformed in the process (e.g. hooked) are additionally conditioned by mechanical anchorage^36^. This observation provides a better understanding of the differences in the rate of descent of σ-ε curve parts in the results from our own research and those from the literature.

In order to confirm the above observations, a comparative analysis of the σ-ε dependence of fiber-reinforced concrete in compression was performed. Table 4 contains selected analytical models describing σ-ε relationships. These models are mostly based on the previously proposed model developed by Carreira and Chu^37^ presented by Eq. (6).

6 $$\sigma ={f}_{c }\frac{\beta \left(\frac{\varepsilon }{{\varepsilon }_{0}}\right)}{\beta -1+{\left(\frac{\varepsilon }{{\varepsilon }_{0}}\right)}^{\beta }}$$

where σ and ε—stresses and strains of the composite, respectively; β—material constant; f_c_—compressive strength of the composite; ε_0_—strains corresponding to the maximum compressive stress.

**Table 4:** Analytical models presenting relationships σ-ε during the compression of concrete reinforced with steel fibers.

Ezeldin and Balaguru^11^	Equation (6) ${f}_{c}={f}_{c}{\prime}+\mathrm{11,232}{V}_{f}\lambda ; \beta =\mathrm{1,093}+0.2429{V}_{f}{\lambda }^{-0.926};$ ${\varepsilon }_{0}={\varepsilon }_{0}{\prime}+1427\cdot 1{0}^{-6}{V}_{f}\lambda$
Someh and Saeki^37,38^	Equation (6) $\beta =\mathrm{1,032}[{f}_{c}\left(1+{V}_{f}\lambda \right){]}^{0.113}; {\varepsilon }_{0}=1.84\cdot 1{0}^{-3}{f}_{c}^{0.147}$
Nataraja et al.^22^	Equation (6) ${f}_{c}={f}_{c}{\prime}+6.9133{V}_{f}\lambda ; \beta =0.5811+0.8155{V}_{f}{\lambda }^{-0.7406};$ ${\varepsilon }_{0}={\varepsilon }_{0}{\prime}+1.92\cdot 1{0}^{-3}{V}_{f}\lambda$
Mansur et al.^5^	Equation (6) dla $0\le \frac{\varepsilon }{{\varepsilon }_{0}}\le 1;$ $\sigma ={f}_{c }\frac{{k}_{1}\beta \left(\frac{\varepsilon }{{\varepsilon }_{0}}\right)}{{k}_{1}\beta -1+{\left(\frac{\varepsilon }{{\varepsilon }_{0}}\right)}^{{k}_{2}\beta }} dla 1<\frac{\varepsilon }{{\varepsilon }_{0}};$ $\beta =\frac{1}{\left[1-\left(\frac{{f}_{c}}{{\varepsilon }_{0}E}\right)\right]}; E=\left(10300-4000{V}_{f}\right){f}_{c}^{0.33} ;$ ${\varepsilon }_{0}=(5\bullet 1{0}^{-4}+7.2\bullet 1{0}^{-4}{V}_{f}\lambda ){f}_{c}^{0.35}$
Baros et al.^10^	$\sigma ={f}_{c }\frac{\frac{\varepsilon }{{\varepsilon }_{0}}}{\left(1-p-q\right)+q\left(\frac{\varepsilon }{{\varepsilon }_{0}}\right)+p{\left(\frac{\varepsilon }{{\varepsilon }_{0}}\right)}^{\frac{1-q}{p}}};$ $q=1-p-\frac{{E}_{1}}{E}; p=1-0.919exp\left(-0.394{W}_{f}\right); {E}_{1}=\frac{{f}_{c }}{{\varepsilon }_{0}};$ $E=21500{\left(\frac{{f}_{c}}{10}\right)}^{0.33}; {W}_{f}=\frac{{w}_{f}}{\rho }; {\varepsilon }_{0}=2.2\cdot 1{0}^{-3}+2\cdot 1{0}^{-4}{W}_{f};$ ${W}_{f}=\frac{{w}_{f}}{\rho }$*;* ρ—gęstość kompozytu [kg/m^3^]; w_f_—zawartość włókien stalowych [kg/m^3^]
Ou et al.^21^	Equation (6) ${f}_{c}={f}_{c}{\prime}+2.35{V}_{f}\lambda ; \beta =0.75({V}_{f}\lambda {)}^{2}-2{V}_{f}\lambda +3.05;$ ${\varepsilon }_{0}={\varepsilon }_{0}{\prime}+7\cdot 1{0}^{-4}{V}_{f}\lambda$
Lee et al.^8^	$\sigma ={f}_{c }\frac{A\left(\frac{\varepsilon }{{\varepsilon }_{0}}\right)}{A-1+{\left(\frac{\varepsilon }{{\varepsilon }_{0}}\right)}^{B}}; A=B=\left(\frac{1}{1+\frac{{f}_{c}}{E{\varepsilon }_{0}}}\right) gdy\frac{\varepsilon }{{\varepsilon }_{0}}\le 1;$ $A=1+0.723({V}_{f}\lambda {)}^{-0.957} gdy \frac{\varepsilon }{{\varepsilon }_{0}}>1;$ $B=\left(\frac{{f}_{c}}{50}\right)\mathrm{0,064}\left(1+0.882{\left({V}_{f}\lambda \right)}^{-0.882}\right)\ge A gdy\frac{\varepsilon }{{\varepsilon }_{0}}>1;$ $E=\left(-367{V}_{f}\lambda +5520\right){f}_{c}^{0.41};$ ${\varepsilon }_{0}=(3\cdot 1{0}^{-4}{V}_{f}\lambda +1.8\cdot 1{0}^{-3}){f}_{c}^{0.12}$

E, E_1_—elastic modulus of the composite.

It should be noted that the models in Table 4 were developed based on tests of concrete modified with steel fibers, which were not short and straight in shape, unlike the fibers used for concrete in this study. Part of the models, i.e. Someh and Saeki^38^, Nataraja et al.^22^, Baros et al.^10^ and Ou et al.^21^, were specified for plain concrete (f_c_ < 50 MPa). However, due to the intention of conducting the broadest possible comparative analyses, they were decided to be considered.

**Figure 11:**
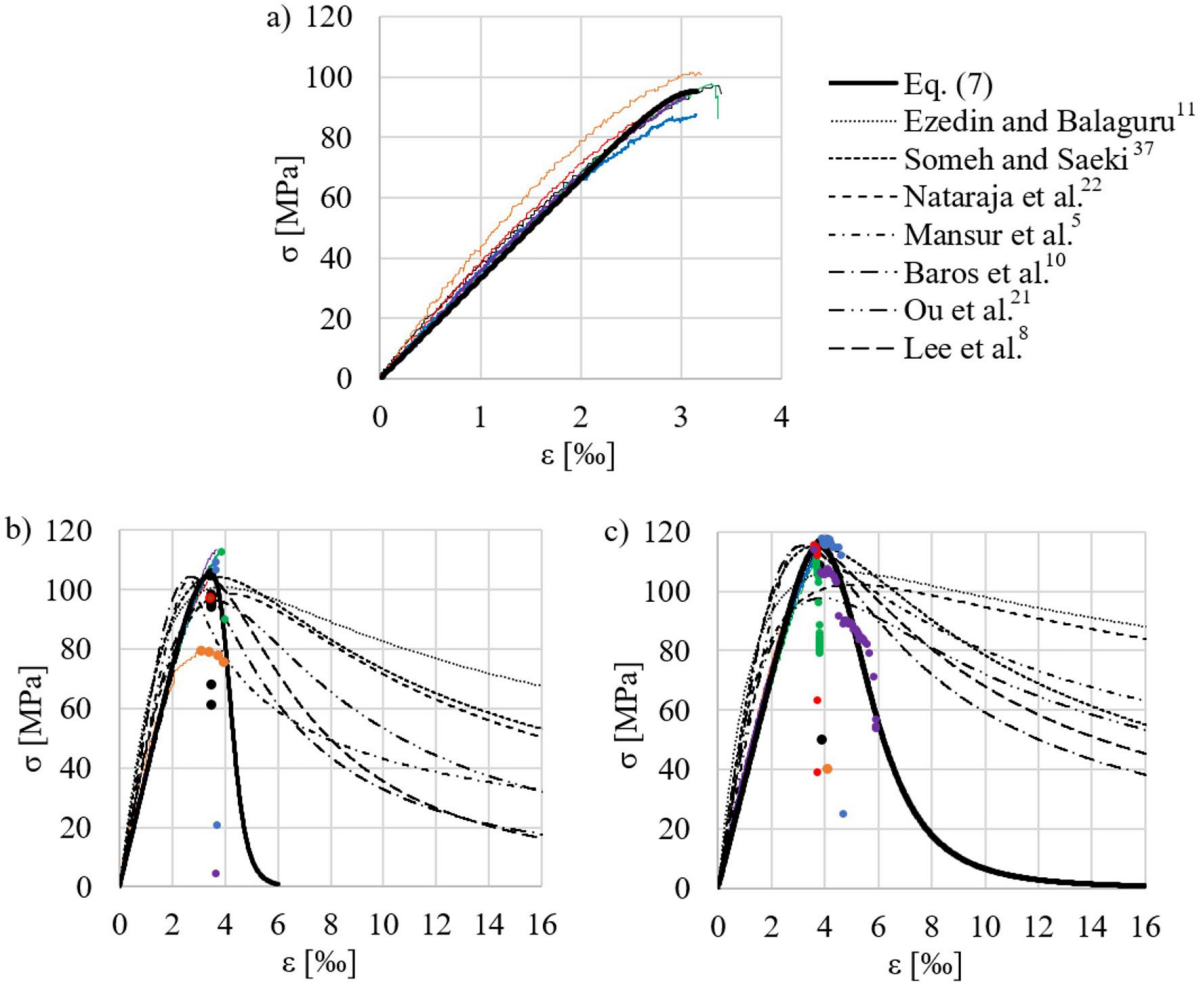
Comparisons of σ-ε relationships of HSC reinforced with short steel fibers inn compression based on analytical models and own experimental tests: (**a**) A-series (without fibers); (**b**) B-series—V_f_λ = 0,49; (**c**) C-series—V_f_λ = 0,98 (sample designations  1;  2;  3;  4;  5;  6, see Table 2;).

Experimental values of the parameters f_c_’ and ε_0_ (arithmetic mean of the results of the series, see Table 2) were used for the analysis—for some models also the parameter f_c_. It was assumed that for the strain ε_0_ the equation ε_0_ = ε^LVDT^ occurs. Figure 11 shows the σ–ε curves from our own tests, as well as the curves described by the equations in Table 4. Comparing the experimental and theoretical results, it can be seen that all the considered σ–ε models overestimate the σ stresses in the descending phase of the curve, hence to describe the σ–ε relationship of HSC reinforced with short fibers, our own analytical model Eq. (7) was proposed, which is also a modification of the proposal by Carreira i Chu^37^.

7 $$\sigma ={f}_{c }\frac{\beta \left(\frac{\varepsilon }{{\varepsilon }_{0}}\right)}{\beta -1+{\left(\frac{\varepsilon }{{\varepsilon }_{0}}\right)}^{\beta }}\left\{\begin{array}{c}\beta ={\beta }_{1} gdy \frac{\varepsilon }{{\varepsilon }_{0}} \le 1\\ \beta ={\beta }_{2} gdy \frac{\varepsilon }{{\varepsilon }_{0}}>1\end{array}\right.$$

where β_1_, β_2_—material constants of the ascending and descending parts of the σ-ε curve, respectively; other designations used as in Eq. (6); f_c_ and ε_0_ defined respectively by Eq. (3 i 4).

**Figure 12:**
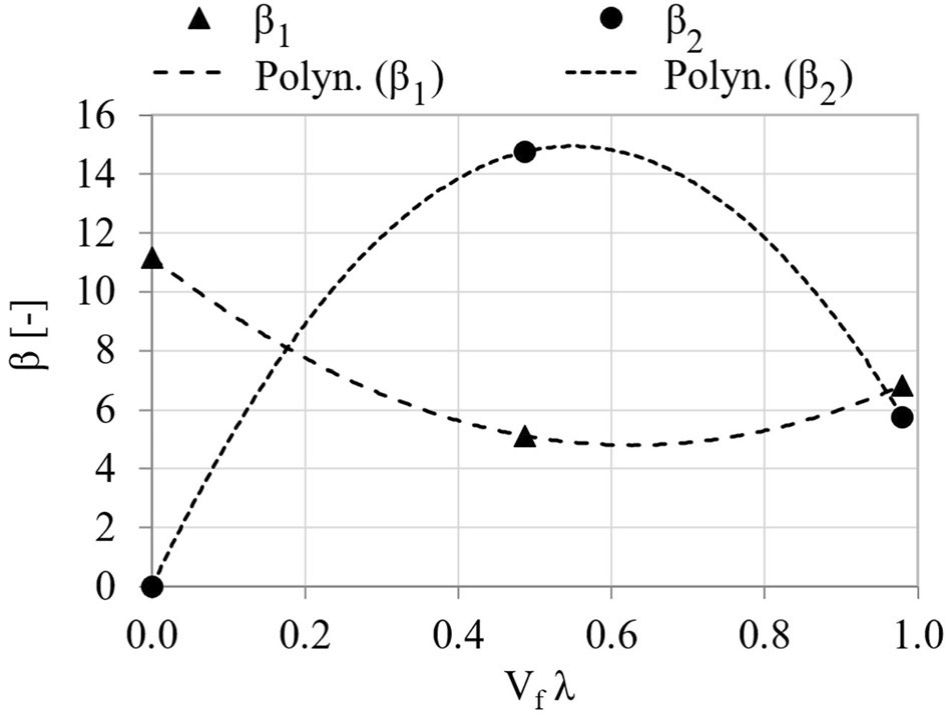
Parameters β_1_ i β_2_ as a function of the degree of fiber reinforcement.

Using the Levenberg–Marquardt estimation method, β parameters of the model (7) were estimated. For HSC with the degree of fiber reinforcement V_f_λ = 0.49 (batch B) parameters β_1_ and β_2_ are 5.12 i 14.76, respectively—with a standard error 0.032 and 8.89. In turn, for the composite with the degree of fiber reinforcement V_f_λ = 0.98 (batch C) parameters β_1_ and β_2_ have values of 6.84 and 5.75 (standard errors—0.045 and 0.418). In the description of the σ-ε relationship of HSC the structural parameters were set as β_1_ = 11.17 (standard error 0.07) and β_2_ = 0. All β parameters of the model are statistically significant except for the β_2_ parameter for batch B, for which a relatively large standard error was observed. This is most likely due to the large scatter in the results from our own tests, as well as the small test sample (the descending part of the σ-ε curve, which is described as points in Fig. 11b and c). This state of affairs was due to the limitation of the possibility of adopting a high sampling frequency of the results and the abrupt manner in which the B-series specimens were destroyed after exceeding the limit load during the test. In other words, the relatively low content of straight, short steel fibers in the concrete slightly affected the occurrence of residual stress in the composite—to a much lesser extent than in the case of the results of the C-series specimens). Comparing the theoretical dependencies of σ–ε with experimental results, it can be concluded that the proposed model approximates the actual σ–ε curves quite well, as shown in Fig. 11. Mean absolute percentage error of the model for the results in batch B and C is 7.1% and 8.4%. The error is larger if the results of the model of the falling part of the σ–ε curve are compared (22.6% and 19.9%, respectively). Figure 12 shows the relationship between the parameters β_1_ and β_2_, and the degree of fiber reinforcement, and presents the regression equations of these parameters Eq. (8). The regression equations extend the state of knowledge in the subject matter^21^, however, due to the number of observations, it is not possible to evaluate them statistically. It is worth noting that extending the study to a larger number of sample series, differing in steel fiber content, would make it possible to generalize the conclusions regarding the fit of the proposed model to the data.

8 $$\begin{aligned} \beta_{1} = & 16.35(V_{f} \lambda )^{2} - 20.39V_{f} \lambda + 11.17 \\ \beta_{2} = & - 0.57(V_{f} \lambda )^{2} - 1.17V_{f} \lambda + 3.15 \\ \end{aligned}$$

## Conclusions

Based on the presented test results of HSC concrete modified with short straight steel fibers and analysis, the following conclusions were made:

- the addition of 1.5% short straight steel fibers to HSC resulted in an increase of: compressive strength by 21%; strain ε_0_ corresponding to maximum compressive stress σ = f_c_ between 14 and 19% (depending on the method of measurement) and toughness ratio (TR) by 20%;
- in the proposed regression equations estimating: compressive strength Eq. (2); strain ε_0_ Eq. (4) and TR (Eq. 5), statistically significant structural parameters were obtained, while the equations’ coefficient of determination values were 0.79, 0.69 and 0.54, respectively;
- the use of the ultrasound method enabled the estimation of compressive strength by means of the regression equation Eq. (3), in which 75% of the variation in the dependent variable was explained by the model;
- the measurement of composite (modified with short straight steel fiber) strain during compression using LVDT, strain gauges, and DIC method gave similar results (Table 2 and Fig. 9);
- the analytical models available in the literature presenting the σ-ε dependence of fiber-reinforced concrete overestimate the results in relation to those obtained from our own experimental studies (HSC with short straight steel fibers);
- the analytical model proposed in the paper Eq. (7) approximates the actual σ-ε curves fairly well (Fig. 11), with an average percentage prediction error of about 8%.

## Data Availability

The data that support the findings of this study are available from the corresponding author upon reasonable request.
