# Author Correction: Effects of the addition of short straight steel fibers on the strength and strains of high-strength concrete during compression

**DOI:** 10.1038/s41598-024-59365-0

**Published:** 2024-04-15

**Authors:** Maciej Kaźmierowski, Roman Jaskulski, Michał Drzazga, Marek Nalepka, Michał Kordasz

**Affiliations:** 1 Department of Civil Engineering, Faculty of Environmental Engineering and Geodesy, Wrocław University of Environmental and Life Sciences, 50-365 Wrocław, Poland; 2 Department of Mechanics and Structural Engineering, Faculty of Civil Engineering and Architecture, Opole University of Technology, 45-061 Opole, Poland; 3 Department of Graphics, Computer Vision and Digital Systems, Faculty of Automatic Control, Electronics and Computer Science, Silesian University of Technology, 44-100 Gliwice, Poland

Correction to: *Scientific Reports* 10.1038/s41598-024-57574-1, published online 24 March 2024

The original version of this Article contained an error in the Funding section.

“The APC is financed by Wrocław University of Environmental and Life Science. This work was supported by the Wrocław University of Environmental and Life Sciences (Poland) as part of the Ph.D. research program “Innovative Scientist”, No N060/0012/2.”

now reads:

“The APC is financed by Wrocław University of Environmental and Life Sciences. This work was supported by the Wrocław University of Environmental and Life Sciences (Poland) as part of the Ph.D. research program “Innovative Scientist”, No N060/0012/21.”

The original Article has been corrected.

